# Downregulation of lncRNA NEAT1 interacts with miR-374b-5p/PGAP1 axis to aggravate the development of osteoarthritis

**DOI:** 10.1186/s13018-023-04147-z

**Published:** 2023-09-11

**Authors:** Feiri Huang, Zhongliang Su, Jie Yang, Xizhen Zhao, Yaozeng Xu

**Affiliations:** 1 Department of Orthopedics, The First Affiliated Hospital of Soochow University, No.188 Shizi Street, Suzhou, 215000 Jiangsu China; 2 Department of Orthopedics, The Third Affiliated Hospital of Shanghai University, The Wenzhou Third Clinical Institute Affiliated to Wenzhou Medical University, Wenzhou, 325000 China

**Keywords:** Osteoarthritis, NEAT1, miR374b-5p, PGAP1, Inflammation, Apoptosis, Cell cycle

## Abstract

**Background:**

Osteoarthritis (OA), characterized by inflammation and articular cartilage degradation, is a prevalent arthritis among geriatric population. This paper was to scrutinize the novel mechanism of long noncoding RNA (lncRNA) NEAT1 in OA etiology.

**Methods:**

A total of 10 OA patients and 10 normal individuals was included in this study. Cell model of OA was built in human normal chondrocytes induced by lipopolysaccharide (LPS). An OA Wistar rat model was established through intra-articular injection of L-cysteine and papain mixtures (proportion at 1:2) into the right knee. Quantitative reverse transcription-polymerase chain reaction was employed to ascertain the expression levels of NEAT1, microRNA (miR)-374b-5p and post-GPI attachment to protein 1 (PGAP1), while dual-luciferase reporter experiments were used for the validation of target relationship among them. Cell cycle and apoptosis were calculated by flow cytometry analysis. CCK-8 assay was done to evaluate the proliferative potentials of chondrocytes. The levels of cell cycle-related proteins (Cyclin A1, Cyclin B1 and Cyclin D2) and pro-apoptotic proteins (Caspase3 and Caspase9) were measured by western blotting. Tumor necrosis factor-alpha (TNF-α), interleukin-1beta (IL-1β) and IL-6 levels were determined via ELISA. Hematoxylin & eosin (HE) Staining was used for pathological examination in OA rats.

**Results:**

Pronounced downregulation of NEAT1 and PGAP1 and high amounts of miR-374b-5p were identified in OA patients, LPS-induced chondrocytes and OA rats. NEAT1 targeted miR-374b-5p to control PGAP1 expression. Loss of NEAT1 or upregulation of miR-374b-5p dramatically accelerated apoptosis, led to the G1/S arrest and promoted the secretion of inflammatory cytokines in LPS-induced chondrocytes, while ectopic expression of PGAP1 exhibited the opposite influences on chondrocytes. Additionally, we further indicated that upregulation of miR-374b-5p attenuated the effects of PGAP1 overexpression on LPS-induced chondrocytes.

**Conclusions:**

Reduced NEAT1 induces the development of OA via miR-374b-5p/PGAP1 pathway. This suggests that the regulatory axis NEAT1/miR-374b-5p/PGAP1 is a novel and prospective target for OA treatment.

## Introduction

Osteoarthritis (OA) is a prevalent arthritis among geriatric population, with chronic joint pain [1]. The clinical symptoms of OA include joint impairment, joint stiffness and pain [2]. It is generally characterized by synovitis and articular cartilage degradation [2]. The roles of pro-inflammatory cytokines and chondrocytes are increasingly being recognized in the pathogenesis of OA. Chondrocytes are involved in maintaining a balanced cartilage turnover and respond to changes induced by cytokines [3]. The loss of chondrocytes reduces the effective turnover of extracellular matrix and cartilage volume, eventually leading to cartilage loss and OA development [4, 5]. Current treatment opinions for OA including bis-phosphonate drugs, non-steroidal anti-inflammatory pills, opioids and steroids, but varying degree of efficacies for OA patients are reported [6–8]. Therefore, potentiating the comprehension of pathogenicity behind the initiation and progression of OA is imperative for exploring novel therapies.

Long noncoding RNAs (lncRNAs) are defined as a category of RNA molecules that are unable to code proteins, which can affect the transcriptional and post-transcriptional expression of genes [9–11]. On account of the different structures and location in cells, lncRNAs are classified into five sub-categories including processed transcripts, sense-intronic transcripts, sense-overlapping transcripts, antisense and long intergenic noncoding RNA (lincRNA) [10]. Evidence is now emerging to suggest that lncRNAs serve as central roles in regulating inflammatory responses and maintaining the dynamic equilibrium of chondrocytes that are recognized as two important drivers of OA cartilage pathology [12–14]. For instance, Pearson et al. conducted a RNAseq analysis and they identified two novel inflammation-associated lincRNAs (CILinc01 and CILinc02) that are abnormally downregulated in OA chondrocytes [15]. Pronounced reduction in CILinc01 and CILinc02 can distinctly enhance the secretion of f interleukin (IL)-6, IL-8 and tumor necrosis factor-alpha (TNF-α) [15]. Xiao et al. uncovered that lncRNA HOTAIRM1-1 is lowly expressed in OA cartilages and at the same time, loss of HOTAIRM1-1 induces chondrocyte apoptosis overtly [16]. Xu et al. established an OA cell model in the presence of IL-1β and found that lncRNA SNHG7 expression is reduced in IL-1β-induced chondrocytes [17]. Moreover, suppression of SNHG7 dramatically induces apoptosis and inflammatory reactions in IL-1β-induced chondrocytes [17]. Nuclear paraspeckle assembly transcript 1 (NEAT1), a nuclear lncRNA located at chromosome 11, plays a vital role in several physiology and pathophysiology processes, such as tumorigenesis [18, 19], organ development [20] and innate immune reactions [21]. Additionally, NEAT1 has been reported to function as a competing endogenous RNA (ceRNA) by competitively binding to microRNAs (miRNAs) to modulate OA progression, such as NEAT1-miR-193a-3p [22], NEAT1-miR-543 [23] and NEAT1-miR-16-5p [24]. It is well-known that the onset and development of OA is a complex biological process referring to multiple regulatory approaches [25]. Whether there are some other regulatory mechanisms underlying NEAT1 in OA progression arouse our great interest.

Some miRNAs have promoting effects in OA. For instance, miR-29b-3p overexpression promotes articular chondrocytes apoptosis and cartilage loss in the knee joint of surgically induced OA rats [26]. miR-335-5p functions as a promoter of chondrocytes apoptosis in vitro [27]. The expression levels of miR-20a in rats with OA patients are positively correlated with inflammatory responses [28]. Additionally, miR-374b-5p exerts obvious pro-inflammatory role in multiple inflammation-related diseases such as microglial activation-caused neuroinflammation [29] and deep vein thrombosis [30]. These findings relate to the effect of miR-374b-5p alone on inflammatory processes. Notably, it remains unclear the role of miR-374b-5p in OA progression and whether the regulatory mechanism of miR-374b-5p is upstream or downstream in OA pathogenesis.

Post-GPI attachment to protein 1 (PGAP1), containing 922 amino-acids, serves as a glycosylphosphatidylinositol (GPI) inositol-deacylase that located at the endoplasmic reticulum membrane [31, 32]. Currently, there are few reports on PGAP1 functions. It has been reported that mutation in PGAP1 is associated with facial dysmorphism, psychomotor retardation and gallbladder cholangiocarcinoma tumorigenesis [33–35]. More importantly, PGAP1 has been reported to be regulated by lncRNA LEMD1-AS1-miR-944 axis to be involved in OA development [36]. Nevertheless, the interactions between miR-374b-5p and PGAP1, as well as the NEAT1/miR-374b-5p/PGAP1 axis in the pathogenesis of OA, are relatively unknown.

The aim of the present study was to investigate a novel molecular mechanism of NEAT1 in OA, and to identify the associations between NEAT1, miR-374b-5p and PGAP1. Through this work, the present study aimed to broaden new horizons of OA pathogenesis and develop a solid foundation for targeted OA therapy.

## Methods

### Ethical statement

Ethical approvals were acquired from the ethics committee of our hospital (approval No.KY-2022–335). All individuals enrolled in this study agreed to sign informed consent and this study conforms to the provisions of the Declaration of Helsinki. For animal experiments, all procedures in rats were executed in compliance with the NIH Guide for the Care and Use of Laboratory Animals and at the same time were approved by the ethics committee of our hospital (approval No.2021ZAASLA45).

### Reagents, cells and animals

DMEM/F12 medium was obtained from Hyclone Technologies (Logan, UT, USA). Fetal bovine serum (FBS) was from Gibco (Grand Island, NY, USA). Penicillin–Streptomycin Solution, radioimmunoprecipitation (RIPA) lysis buffer, bicinchoninic acid (BCA) protein assay Kit and enhanced chemiluminescence (ECL) detection Kit were available from Beyotime Biotechnology (Shanghai, China). Propidium iodide/RNase A solution, lipopolysaccharide (LPS), papain and L-cysteine were bought from Sigma-Aldrich (St. Louis, MO, USA). Invitrogen (Carlsbad, CA, USA) provided transfection reagent Lipofectamine 3000 and Trizol reagent. Dojindo Molecular Technologies (Kumamoto, Japan) provided CCK-8 solution was used for the measurement of cell viability. Annexin V-FITC/PI Apoptosis Detection Kit was obtained from Vazyme (Nanjing, China). Hifair® II 1st Strand cDNA Synthesis SuperMix and Hieff® qPCR SYBR Green Master Mix were obtained from Yeasen Biotechnology (Shanghai, China). For western blotting, primary antibodies Caspase3, Caspase9, Cyclin B1, and Cyclin D2, and the horseradish peroxidase (HRP)-conjugated secondary antibody were got from Proteintech (Wuhan, China); primary antibody Cyclin A1 was from Abcam (Cambridge, UK); primary antibody GAPDH was available from ATaGenix (Wuhan, China). Meanwhile, the hematoxylin & eosin (HE) Staining Kit was also from Abcam. From Promega (Madison, WI, USA), the Dual-Luciferase Reporter Assay System Kit was available. NEAT1-small interfering RNA (siRNA)-1/-2/-3 and the negative control (NC siRNA), overexpression of PGAP1 (OE-PGAP1) and the corresponding negative control (OE-NC), as well as miR-374b-5p mimic/ NC mimic were synthesized by Genomeditech (Shanghai, China). The Phusion Site-Directed Mutagenesis Kit was from Thermo Fisher Scientific (Waltham, MA, USA). Human TNF-α, IL-1β and IL-6 ELISA Kits, and rat TNF-α, IL-1β and IL-6 ELISA Kits were purchased from ImmunoWay Biotechnology (Plano, TX, USA). Human normal chondrocytes were obtained From Procell (Wuhan, China). Male Wistar rats weighing 280–320 g (3-month-old) were purchased from Vital River Laboratory Animal Technology (Pinghu, China).

### OA samples collection

A total of 10 OA patients (5 males and 5 females; age range, 52–70 years; mean age, 60.5 ± 6.4) was enrolled in our hospital. The diagnostic criteria for OA were based on the American College of Rheumatology standards [37]. The inclusion criteria were: (1) newly diagnosed OA cases; (2) no therapies initiated; (3) fully understood the experimental protocol. The exclusion criteria were: (1) patients complicated with other clinical disorders, such as chronic inflammatory diseases other than OA; (2) patients who were treated within 3 months before admission. The patients according with the above conditions were chosen in this study and eventually the sample size was 10. Meanwhile, ten normal individuals (5 males and 5 females; age range, 48–66 years; mean age, 56.7 ± 6.4) with femoral neck fracture were served as the controls and they had no history of rheumatoid arthritis or OA. OA cartilage tissues were isolated from OA patients underwent total knee arthroplasty, while the normal articular cartilage was obtained from the normal individuals who received the total hip replacement surgery. The collected samples were immediately stored at − 80 °C until used.

### Cell culture and treatment

Human normal chondrocytes were grown in DMEM/F12 containing 10% FBS and 1% Penicillin–Streptomycin Solution, with the environmental conditions of 5% CO_2_ at 37 °C. The second passage chondrocytes at 85% confluency were used in subsequent experiments. To mimic OA in cellular level, LPS (5 µg/mL) was used to treat normal chondrocytes for 12 h [38].

### Cell transfection

Human normal chondrocytes (6 × 10^5^ cells/well) were co-transfected with NEAT1-siRNA-1/-2/-3, NC siRNA, OE-PGAP1, OE-NC, miR-374b-5p mimic or NC mimic (all, 50 nM), respectively, for 48 h with the aid of Lipofectamine 3000. Chondrocytes without transfection were served as the controls. After that, chondrocytes were gathered and the gene expression levels were detected through quantitative reverse transcription-polymerase chain reaction (qRT-PCR).

### CCK-8 assay

Chondrocytes at a density of 4 × 10^3^ cells/well were plated into 96-well plates, following culturing for 12, 24, 36 and 48 h, respectively. Thereafter, CCK-8 solution was added to each well to incubate for 2 h. The optical density at 450 nm was measured by a microplate reader (DR-3518G, Hiwell Diatek, Wuxi, China).

### Flow cytometry analysis

For the cell cycle assay, chondrocytes (1 × 10^5^) were initially fixed with 75% ethanol for 1 h. After centrifugation at 1500*g* for 5 min, cells were re-suspended in phosphate buffered saline (PBS) and incubated with Propidium iodide/RNase A solution in the dark for 30 min. The frequencies of cells in G1, S and G2 phases were analyzed by FACSCalibur (BD Biosciences, San Jose, CA, USA). For the apoptosis assay, chondrocytes were re-suspended in binding buffer, and then stained with Annexin V-FITC and PI for 20 min in the dark. The apoptotic rate was measured by FACSCalibur (BD Biosciences).

### Western blotting analysis

Proteins were extracted from chondrocytes using RIPA lysis buffer and then quantified via a BCA protein assay Kit. Subsequently, protein samples (40 μg protein/lane) were fractionated by 10% sodium dodecyl sulfate–polyacrylamide gel electrophoresis, with electrophoresis voltage 80 V transferring to 120 V by wet transfer. The proteins were transferred onto polyvinylidene fluoride membranes with transferring voltage of 100 mV for 45–70 min. After blocking with 5% skimmed milk-Tris-buffered saline Tween (TBST) for 2 h at 25 °C, the membranes were incubated overnight with corresponding primary antibodies Caspase3 (dilution in 1:500), Caspase9 (dilution in 1:600), Cyclin A1 (dilution in 1:1000), Cyclin B1 (dilution in 1:1000), Cyclin D2 (dilution in 1:1000) and GAPDH (dilution in 1:5000) at 4 °C. After the membranes were washed with TBST three times, the HRP-conjugated secondary antibody (dilution in 1:5000) was added and incubated at 25 °C for 1 h. GAPDH was selected as the internal reference. Blot signals were measured via an ECL detection Kit and the relative protein expression levels were semi-quantified using the ChemiDoc XRS system (Bio-Rad Laboratories, Inc.).

### Target prediction

The miRNA targets of NEAT1 were predicted using Starbase software (http://starbase.sysu.edu.cn/), and 144 targets were predicted. Among these miRNA targets, miR-374b-5p was selected due to the unknown regulatory relationship with NEAT1 in OA. In addition, the mRNA targets of miR-374b-5p were predicted using Starbase software, and 1248 targets were predicted. PGAP1 was selected for the following assays due to its important role in OA and the unknown regulatory relationship with miR-374b-5p.

### Dual-luciferase reporter experiments

The partial sequence of the 3′‑untranslated region (UTR) NEAT1 and the 3′‑UTR of PGAP1 containing the putative binding sites of miR‑374b‑5p were synthetized and obtained from Sangon Biotech (Shanghai, China). Subsequently, the sequences were cloned into the pGL3 vector to construct wild‑type (WT) reporter vectors for NEAT1 and PGAP1. The mutant (MUT) NEAT1 and PGAP1‑3′‑UTR sequences containing the putative binding sites of miR‑374b‑5p were created using the Phusion Site-Directed Mutagenesis Kit, according to the manufacturer's protocol, and cloned into pGL3 vectors to construct MUT reporter vectors for NEAT1 and PGAP1. The ratio of vector and insert was 1:5. The reporter plasmids (10 nM) together with miR-374b-5p mimic or NC mimic (10 nM) were introduced into chondrocytes (5 × 10^3^ cells/well) using Lipofectamine 3000. Dual-Luciferase Reporter Assay System Kit was utilized to determine the relative luciferase activities. Firefly luciferase activity was normalized to *Renilla* luciferase activity.

### OA rat model establishment

A total of 10 male Wistar rats were housed (5 rats/cage) under standard management conditions. Room temperature and humidity were maintained at 20–24 °C and at 50–60%, respectively. The light cycle was fixed at 12 h and the animals were fed a standard rat diet with water ad libitum. The OA rat model was established as previously described [39]. Briefly, Wistar rats were separated into 2 groups randomly: the sham and model groups, with 5 rats in each group. Subsequently, 0.03 mol/L L-cysteine and 4% papain solution were mixed at proportion of 1:2 in volume and allowed to stand for 0.5 h. Following anesthetization with pentobarbital sodium (50 mg/kg), rats in the model group were given the mixtures (20 μL) into the right knee joint via intra-articular injection at 1st, 4th and 7th day, respectively, while rats in the sham group were injected with the equal amount of normal saline. Four weeks after injection, the rats were euthanized and underwent knee amputation. The articular cartilage was obtained for HE staining and qRT-PCR analysis.

### HE staining

Rat articular cartilage was first fixed in 4% paraformaldehyde at room temperature for 2 days and placed in ethylenediaminetetraacetic acid disodium salt (EDTA-2Na) for 8 weeks at room temperature. Tissues were sequentially placed in 50, 70, 80, 95, 100% ethanol to complete dehydration for 45 min at room temperature for each gradient. The embedding frame containing the tissue was then placed in xylene I and xylene II in sequence, and placed in a 65 °C oven for 1 h each. Finally, the dissolved paraffin was introduced into the embedding frame, and after the wax block was completely solidified, the embedding frame was disassembled and the wax block taken out. Paraffin blocks were cut into 4-µm serial sections for standard HE staining. In brief, after the paraffin sections were baked in a 65 °C oven for 30 min, they were placed at room temperature with xylene I and xylene II for 5 min each. Then rehydrate with graded ethanol of 100, 95, 85 and 75% for 3 min. Then, a series of operations such as hematoxylin staining (5 min), differentiation solution (2 min) and eosin staining (1 min) were used, and finally dehydration and mounting were performed. Images were acquired under an optical microscope (OLYMPUS BX53; Olympus, Japan; magnification × 400).

### RNA extraction and qRT-PCR

Total RNA from cartilage tissues of OA patients (100 mg), articular cartilage of rats (50 mg) or chondrocytes (2.5 × 10^6^) was extracted with the aid of Trizol reagent. RNA purity was measured using the NanoDrop (Peqlab Biotechnologie GmbH, Erlangen, Germany). The OD260/280 ratio was used as indicator for RNA purity. A ratio higher than 1.8 was regarded as suitable for gene expression measurements. To determine the expression levels of NEAT1, miR-374b-5p and PGAP1, total RNA (1 µg) was firstly employed into cDNA via a Hifair® II 1st Strand cDNA Synthesis SuperMix Kit at 42 °C for 45 min. Subsequently, PCR analysis was performed using Hieff® qPCR SYBR Green Master Mix on an ABI 7900 Real-Time PCR System (Applied Biosystems, Foster City, CA, USA). The thermocycling conditions were as follows: initial denaturation for 10 min at 95 °C; 40 cycles of 95 °C for 15 s and 60 °C for 30 s; and final extension for 1 min at 60 °C. Gene expression was calculated with 2^−ΔΔCt^ methods, with the controls of GAPDH (for NEAT1 and PGAP1) and U6 (for miR-374b-5p). We designed the primers using Primer-BLAST (http://www.ncbi.nlm.nih.gov/tools/primer-blast). MFEprimer-2.0 (https://github.com/quwubin/MFEprimer/wiki/Manual/) was used to perform specificity checking. Primer sequences used were listed in Table 1.

**Table 1:** Real-time PCR Primer synthesis list

Gene	Sequences	
Human NEAT1	Forward	5′–GCTACAAGGTGGGGAAGACT–3′
	Reverse	5′–AGTCTGACGCCCATCTTTCA–3′
Hsa-miR-374b-5p	Forward	5′–GCGCGATATAATACAACCTGC–3′
	Reverse	5′–AGTGCAGGGTCCGAGGTATT–3′
Human PGAP1	Forward	5′–ACAGGCTCCATCTTCCACAG–3′
	Reverse	5′–ACGACATAAGCAGGAAGAGC–3′
Human GAPDH	Forward	5′–TCAAGAAGGTGGTGAAGCAGG–3′
	Reverse	5′–TCAAAGGTGGAGGAGTGGGT–3′
Human U6	Forward	5′–CGCTTCGGCAGCACATATAC–3′
	Reverse	5′–AAATATGGAACGCTTCACGA–3′

### ELISA

According to vendors’ instructions, the levels of TNF-α, IL-1β and IL-6 in chondrocytes or the serum of rats were determined via the corresponding commercial Kits.

### Statistical analysis

Data were presented as means ± standard deviation (SD). Based on SPSS 23.0 software (SPSS, Chicago, IL, USA). Student’s *t*-test and One-way ANOVA (followed by Tukey's multiple comparisons test) were applied for comparison. The significant difference was considered when *P* < 0.05.

## Results

### Silencing of NEAT1 promotes apoptosis and inflammatory responses in LPS-induced chondrocytes

**Fig. 1:**
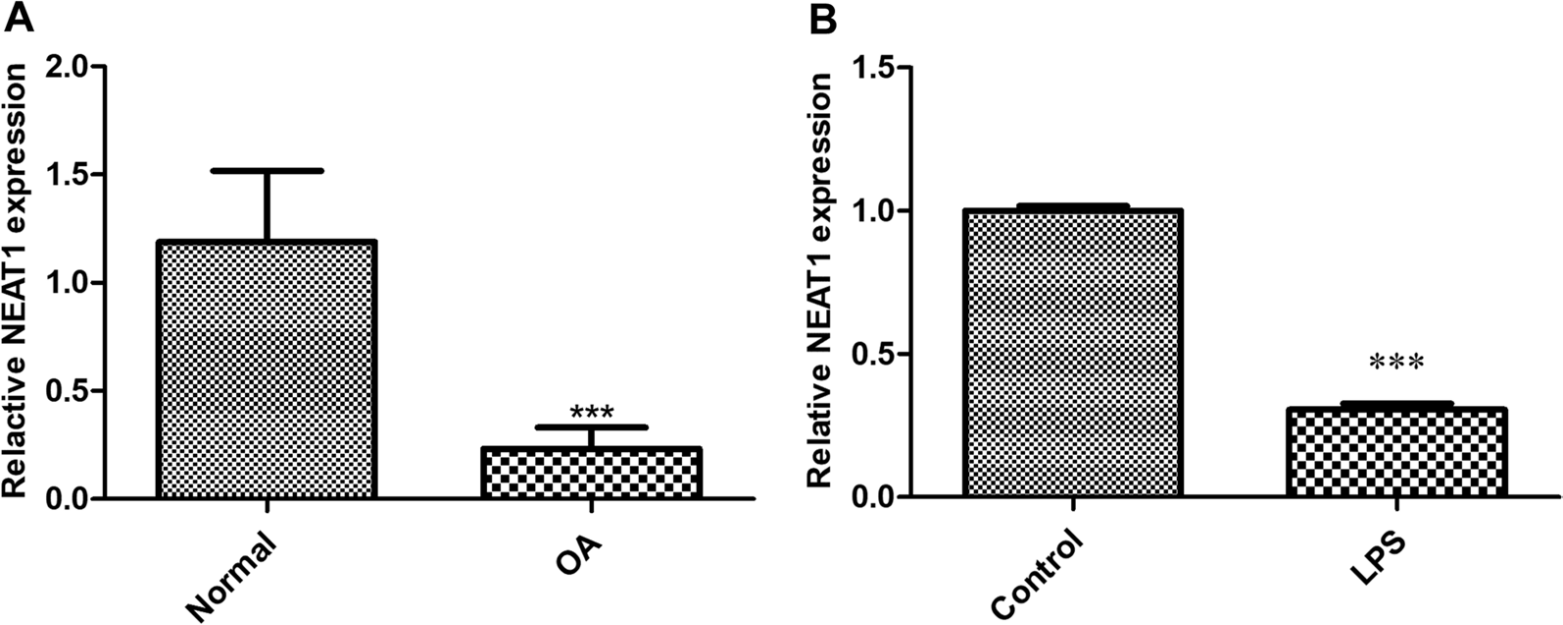
Pronounced downregulation of NEAT1 is observed in OA patients and LPS-induced chondrocytes. **A** The expression of NEAT1 in OA cartilage tissues (n = 10) and normal cartilage tissues (n = 10) was detected via qRT-PCR. ***P < 0.001 vs. normal. **B** The expression of NEAT1 in LPS-induced chondrocytes was detected via qRT-PCR. ***P < 0.001 vs. control. Error bars represented as the means ± standard deviation (SD). Data were all analyzed by Student’s *t*-test

**Fig. 2:**
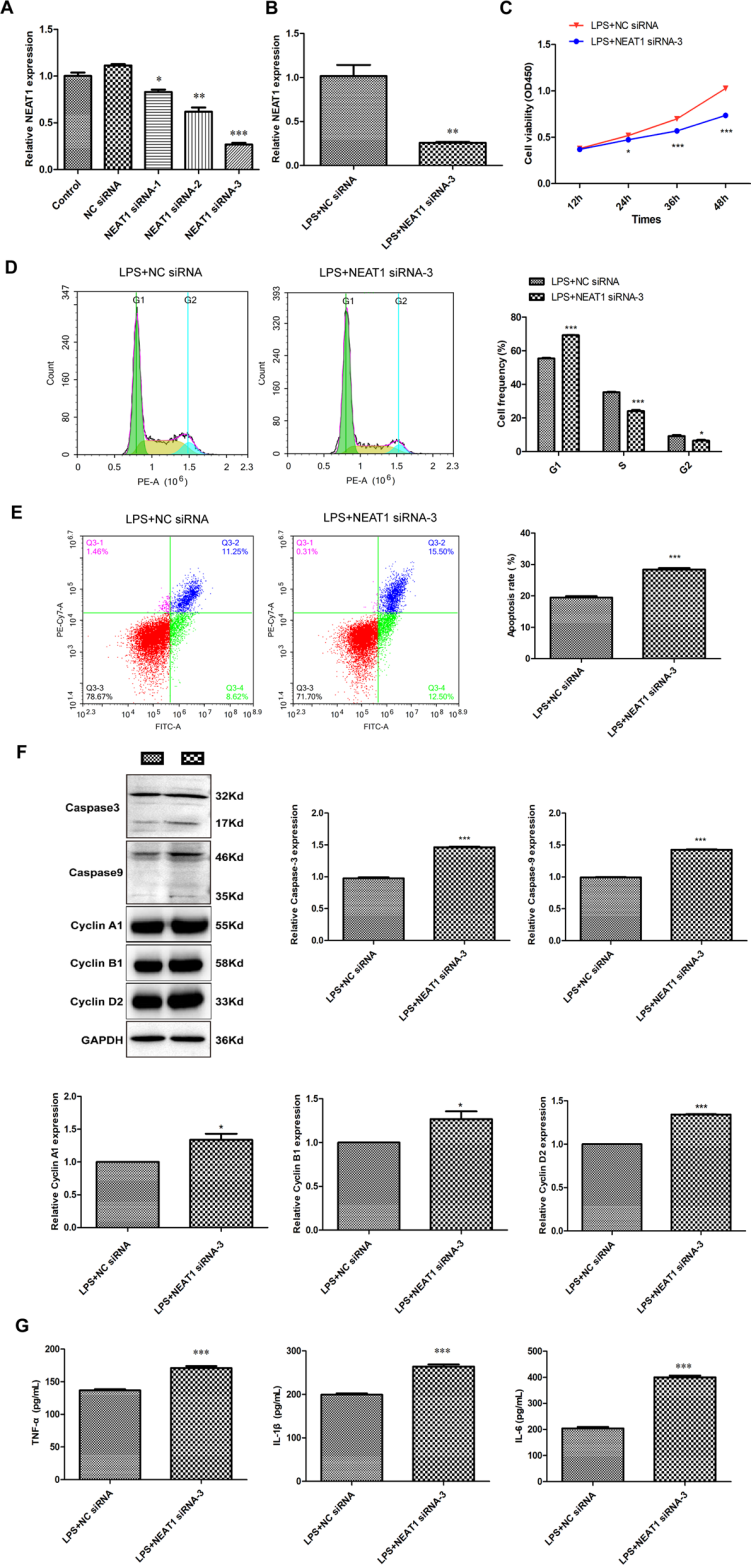
Silencing of NEAT1 promotes apoptosis and inflammatory responses in LPS-induced chondrocytes. **A** The expression of NEAT1 in chondrocytes after transfection with NEAT1 siRNA-1/-2/-3 or NC siRNA was detected via qRT-PCR (analyzed by One-way ANOVA followed by Tukey's multiple comparisons test). *P < 0.05, **P < 0.01, ***P < 0.001 vs. NC siRNA. **B** The expression of NEAT1 in LPS-induced chondrocytes after transfection with NEAT1 siRNA or NC siRNA was detected via qRT-PCR (analyzed by Student’s *t*-test). **P < 0.01 vs. LPS + NC siRNA. **C** The viability of LPS-induced chondrocytes transfected with NEAT1 siRNA/NC siRNA was measured by CCK-8 assay (analyzed by Student’s *t*-test). *P < 0.05, ***P < 0.001 vs. LPS + NC siRNA. Cell frequency (**D**) and apoptosis rate (**E**) of LPS-induced chondrocytes transfected with NEAT1 siRNA/NC siRNA were calculated via flow cytometry analysis (analyzed by Student’s *t*-test). *P < 0.05, ***P < 0.001 vs. LPS + NC siRNA. **F** The protein levels of Caspase3, Caspase9, Cyclin A1, Cyclin B1 and Cyclin D2 in LPS-induced chondrocytes transfected with NEAT1 siRNA/NC siRNA were determined via western blotting (analyzed by Student’s *t*-test). *P < 0.05, ***P < 0.001 vs. LPS + NC siRNA. **G** The levels of TNF-α, IL-1β and IL-6 in LPS-induced chondrocytes transfected with NEAT1 siRNA/NC siRNA were determined via ELISA (analyzed by Student’s *t*-test). ***P < 0.001 vs. LPS + NC siRNA. Error bars represented as the means ± standard deviation (SD)

The expression of NEAT1 in OA patients was determined via qRT-PCR, indicating that NEAT1 was lowly expressed in OA cartilage tissues compared to that of normal cartilage (Fig. 1A, *P* < 0.001). As shown in Fig. 1B, we further demonstrated that in LPS-induced chondrocytes, pronounced reduction in NEAT1 was observed (*P* < 0.001). To probe the effects of NEAT1 on OA progression in vitro, NEAT1 siRNA-1, NEAT1 siRNA-2, or NEAT1 siRNA-3 was firstly introduced into chondrocytes. As indicated in Fig. 2A, NEAT1 had the lowest expression level in chondrocytes when transfected with NEAT1 siRNA-3 (*P* < 0.001). Therefore, we selected NEAT1 siRNA-3 for the succeeding tests. Then, we further demonstrated that in LPS-induced chondrocytes, NEAT1 expression was overtly reduced after NEAT1 siRNA transfection (Fig. 2B, *P*  < 0.01). The effect of NEAT1 silencing on cell viability was then explored in LPS-induced chondrocytes. CCK-8 assay showed that transfection of NEAT1 siRNA significantly repressed the viability of chondrocytes (Fig. 2C, *P* < 0.05). The influence of NEAT1 silencing on cell cycle was further studied in LPS-induced chondrocytes due to its inhibiting effect on cell viability. Compared to the LPS + NC siRNA group, the cell frequency of G1 phase was raised, while the cell frequency of S phase was reduced in the LPS + NEAT1 siRNA group (Fig. 2D, *P*  < 0.001), suggesting that the downregulation of NEAT1 led to the G1/S arrest in LPS-induced chondrocytes. Flow cytometry analysis demonstrated that in the presence of LPS, the apoptosis rate was elevated in NEAT1 siRNA-transfected chondrocytes relative to that in NC siRNA-transfected cells (Fig. 2E, *P *< 0.001). Western blotting was carried out to further verify by checking pro-apoptotic proteins (Caspase3 and Caspase9) and cell cycle-related proteins (Cyclin A1, Cyclin B1 and Cyclin D2). As manifested in Fig. 2F, the protein levels of Caspase3, Caspase9, Cyclin A1, Cyclin B1 and Cyclin D2 were distinctly elevated in LPS-induced chondrocytes transfected with NEAT1 siRNA (*P* < 0.05). Additionally, we further indicated that knockdown of NEAT1 promoted the secretion of inflammatory cytokines (TNF-α, IL-1β and IL-6) in LPS-induced chondrocytes (Fig. 2G, *P*  < 0.001).

### NEAT1 binds to miR-374b-5p

**Fig. 3:**
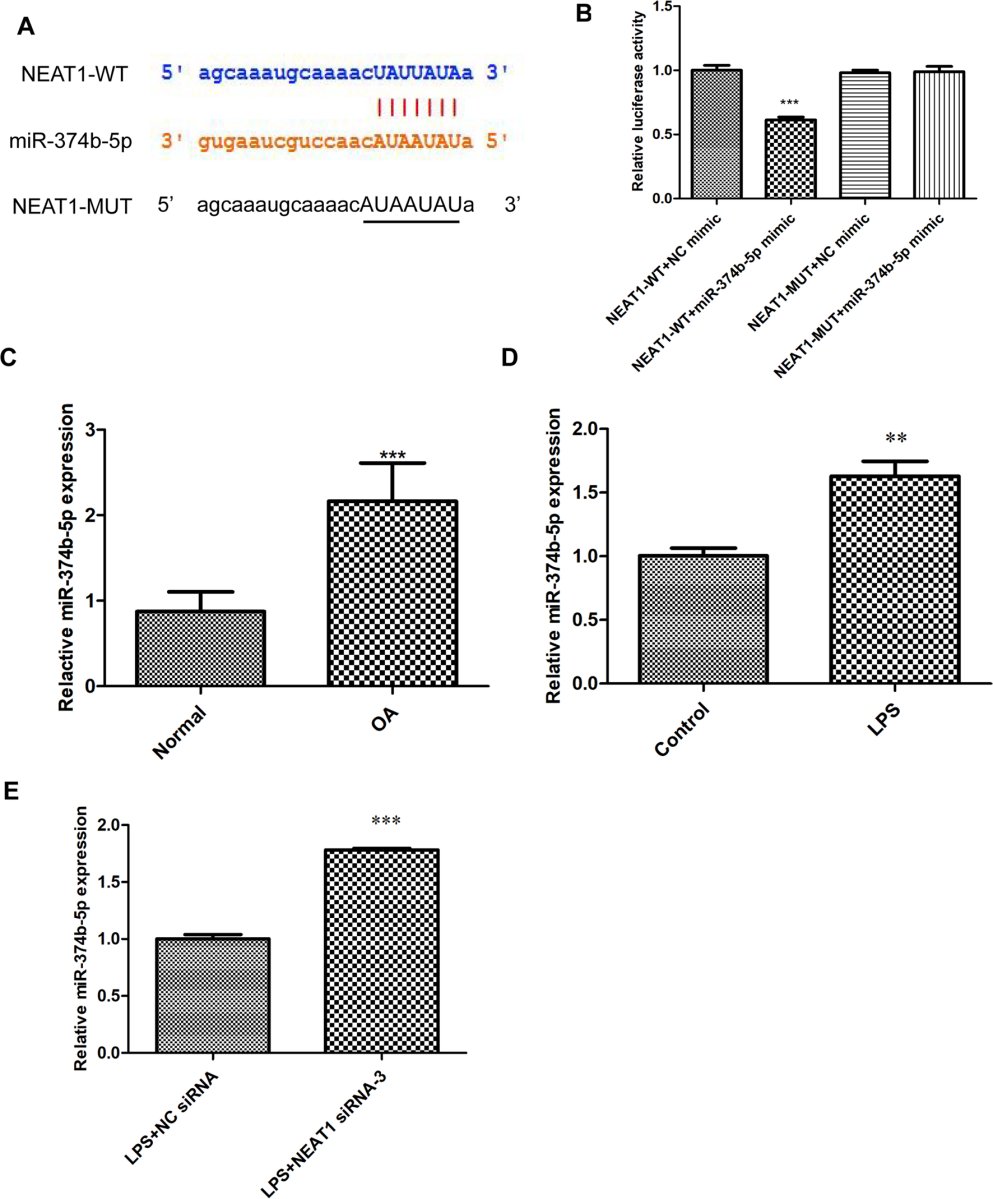
NEAT1 binds to miR-374b-5p. **A** StarBase showed the predicted binding site of NEAT1 and miR-374b-5p. **B** The luciferase activities in chondrocytes co-transfected with pGL3-NEAT1-WT/pGL3-NEAT1-MUT and miR-374b-5p mimic/NC mimic were determined by dual-luciferase reporter assay (analyzed by One-way ANOVA followed by Tukey's multiple comparisons test). ***P < 0.001 vs. NEAT1-WT + NC mimic. **C** The expression of miR-374b-5p in OA cartilage tissues (n = 10) and normal cartilage tissues (n = 10) was detected via qRT-PCR (analyzed by Student’s *t*-test). ***P < 0.001 vs. normal. **D** The expression of miR-374b-5p in LPS-induced chondrocytes was detected via qRT-PCR (analyzed by Student’s *t*-test). **P < 0.01 vs. control. **E** The expression of miR-374b-5p in LPS-induced chondrocytes after transfection with NEAT1 siRNA or NC siRNA was detected via qRT-PCR (analyzed by Student’s *t*-test). ***P < 0.001 vs. LPS + NC siRNA. Error bars represented as the means ± standard deviation (SD)

Starbase software predicts a latent NEAT1 and miR-374b-5p binding site, as shown in Fig. 3A. Dual-luciferase reporter experiments showed that compared to those transfected with NEAT1-WT/NC mimic, transfection of NEAT1-WT/miR-374b-5p mimic dramatically suppressed the luciferase activities in chondrocytes (Fig. 3B, *P*  < 0.001), whereas there were no statistical changes across those introduced with NEAT1-MUT/miR-374b-5p mimic and NEAT1-MUT/NC mimic (Fig. 3B). Moreover, it was demonstrated that miR-374b-5p expression was significantly elevated in OA cartilage tissues (Fig. 3C, *P*  < 0.001) and LPS-induced chondrocytes (Fig. 3D, *P*  < 0.01), compared to the corresponding NCs. Additionally, we further found that miR-374b-5p expression could be increased by NEAT1 siRNA transfection (Fig. 3E, *P*  < 0.001).

### MiR-374b-5p upregulation accelerates apoptosis and inflammatory reactions in LPS-induced chondrocytes

**Fig. 4:**
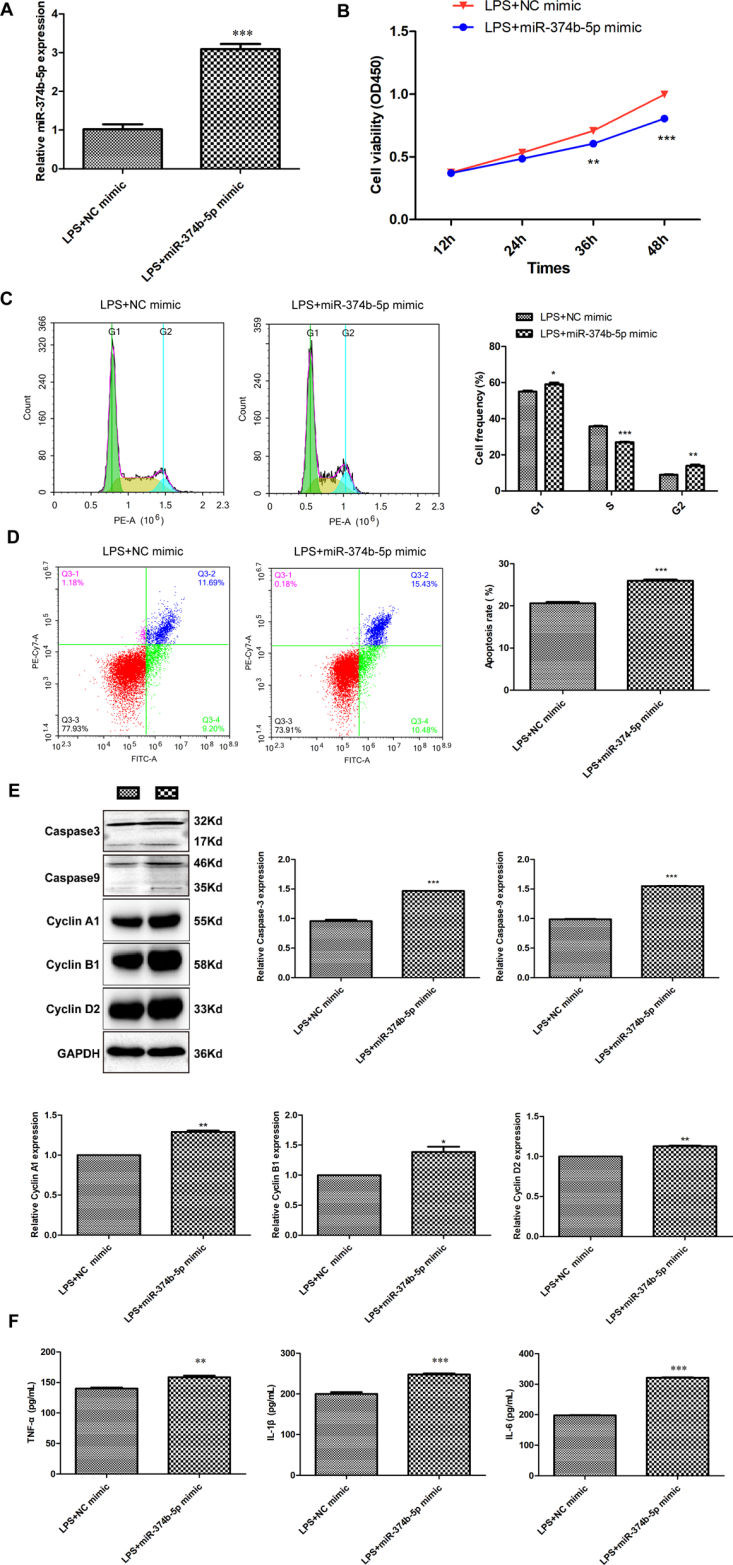
MiR-374b-5p upregulation accelerates apoptosis and inflammatory reactions in LPS-induced chondrocytes. **A** The expression of miR-374b-5p in LPS-induced chondrocytes after transfection of miR-374b-5p mimic or NC mimic was detected by qRT-PCR. ***P < 0.001 vs. LPS + NC mimic. **B** The viability of LPS-induced chondrocytes transfected with miR-374b-5p mimic/NC mimic was measured by CCK-8 assay. **P < 0.01, ***P < 0.001 vs. LPS + NC mimic. Cell frequency (**C**) and apoptosis rate (**D**) of LPS-induced chondrocytes transfected with miR-374b-5p mimic/NC mimic were calculated via flow cytometry analysis. *P < 0.05, **P < 0.01, ***P < 0.001 vs. LPS + NC mimic. **E** The protein levels of Caspase3, Caspase9, Cyclin A1, Cyclin B1 and Cyclin D2 in LPS-induced chondrocytes transfected with miR-374b-5p mimic/NC were determined via western blotting. *P < 0.05, **P < 0.01, ***P < 0.001 vs. LPS + NC mimic. **F** The levels of TNF-α, IL-1β and IL-6 in LPS-induced chondrocytes transfected with miR-374b-5p mimic/NC were determined via ELISA. **P < 0.01, ***P < 0.001 vs. LPS + NC mimic. Error bars represented as the means ± standard deviation (SD). Data were all analyzed by Student’s *t*-test

To ascertain the role of miR-374b-5p in the progression of OA in vitro, miR-374b-5p mimic or NC mimic was further introduced into LPS-induced chondrocytes (Fig. 4A, *P* < 0.001). In functional analysis, it was revealed that the viability of LPS-induced chondrocytes transfected with miR-374b-5p mimic was significantly repressed by contrast to those transfected with NC mimic (Fig. 4B,  *P* < 0.01). Flow cytometry showed that upregulation of miR-374b-5p caused G1 arrest in LPS-induced chondrocytes (Fig. 4C, *P *< 0.05). Meanwhile, significantly increased apoptosis rate was observed in LPS-induced chondrocytes transfected with miR-374b-5p mimic relative to those transfected with NC mimic (Fig. 4D, *P* < 0.001). As illustrated in Fig. 4E, high expression of miR-374b-3p not only increased the protein levels of Caspase3 and Caspase9, but also remarkably elevated the levels of Cyclin A1, Cyclin B1 and Cyclin D2 (*P* < 0.05). In addition, based on the results of ELISA, we found that compared with LPS-induced chondrocytes transfected with NC mimic, pronounced increase in TNF-α, IL-1β and IL-6 was determined in those transfected with miR-374b-5p mimic (Fig. 4F, *P* < 0.01).

### PGAP1 is a target gene of miR-374b-5p

**Fig. 5:**
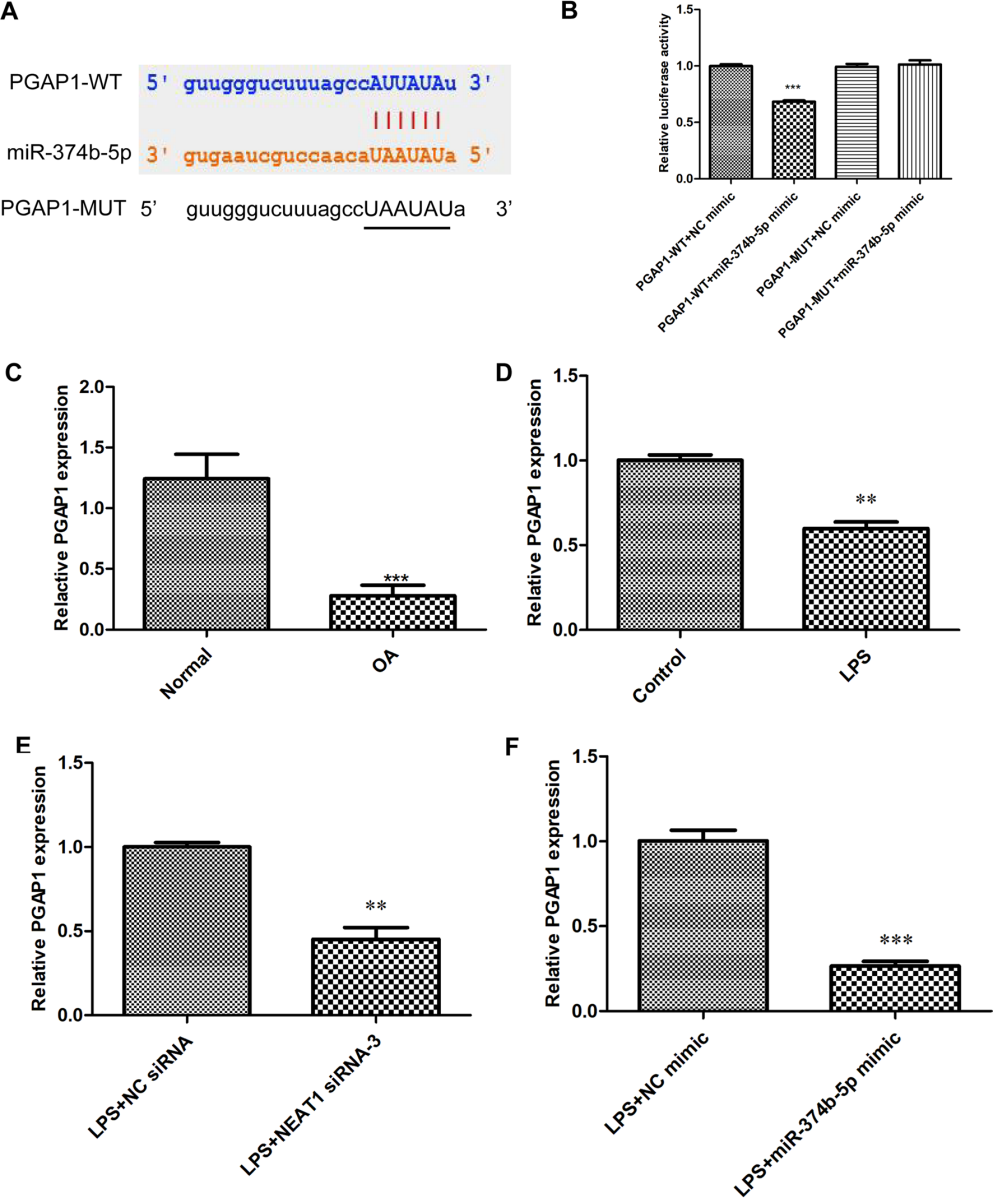
PGAP1 is a target gene of miR-374b-5p. **A** StarBase showed the predicted binding site between miR-374b-5p and PGAP1. **B** The target relationship between miR-374b-5p and PGAP1 was verified by dual-luciferase reporter assay in chondrocytes (analyzed by One-way ANOVA followed by Tukey's multiple comparisons test). ***P < 0.001 vs. PGAP1-WT + NC mimic. **C** The expression of PGAP1 in OA cartilage tissues (n = 10) and normal cartilage tissues (n = 10) was detected via qRT-PCR (analyzed by Student’s *t*-test). ***P < 0.001 vs. normal. **D** The expression of PGAP1 in LPS-induced chondrocytes was detected via qRT-PCR (analyzed by Student’s *t*-test). **P < 0.01 vs. control. **E** The expression of PGAP1 in LPS-induced chondrocytes after transfection with NEAT1 siRNA or NC siRNA was detected via qRT-PCR (analyzed by Student’s *t*-test). **P < 0.01 vs. LPS + NC siRNA. **F** The expression of PGAP1 in LPS-induced chondrocytes after transfection with miR-374b-5p mimic or NC mimic was detected via qRT-PCR (analyzed by Student’s *t*-test). ***P < 0.001 vs. LPS + NC mimic. Error bars represented as the means ± standard deviation (SD)

Also based on the prediction of Starbase software, an underlying miR-374b-5p and PGAP1 binding site was delineated in Fig. 5A. As exhibited in Fig. 5B, we performed luciferase reporter experiments and uncovered that miR-374b-3p mimic could visibly decrease the luciferase activity of PGAP1-WT reporter vector in chondrocytes (*P* < 0.001). Meanwhile, the luciferase activities between the PGAP1-MUT/miR-374b-5p mimic and PGAP1-MUT/NC mimic groups showed no significant differences. In OA cartilage tissues and LPS-induced chondrocytes, PGAP1 mRNA expression was found to be reduced (Fig. 5C–D, *P * < 0.01). Additionally, we further found that the expression of PGAP1 in LPS-induced chondrocytes could be decreased by transfection of NEAT1 siRNA (Fig. 5E, *P * < 0.01) or miR-374b-5p (Fig. 5F, *P * < 0.001).

### MiR-374b-5p regulates PGAP1 expression to affect the apoptosis and inflammation in LPS-induced chondrocytes

**Fig. 6:**
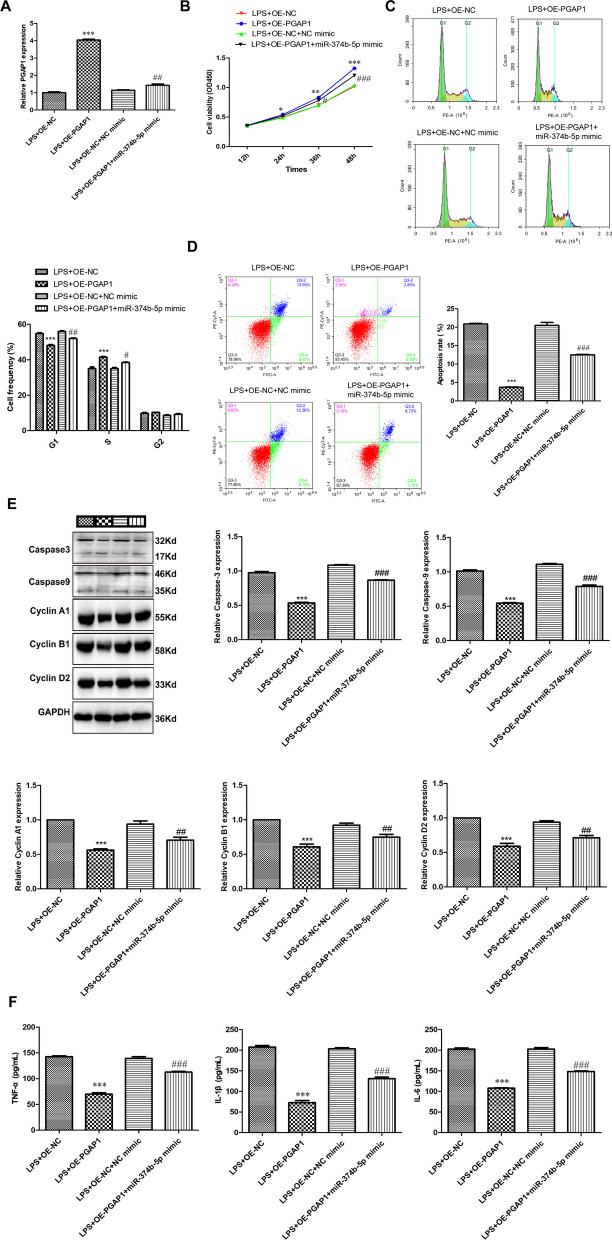
MiR-374b-5p regulates PGAP1 expression to affect the apoptosis and inflammation in LPS-induced chondrocytes. **A** The expression of PGAP1 in LPS-induced chondrocytes transfected with OE-NC, OE-PGAP1, OE-NC + NC mimic or OE-PGAP1 + miR-374b-5p mimic was detected via qRT-PCR. **B** The viability of LPS-induced chondrocytes transfected with OE-NC, OE-PGAP1, OE-NC + NC mimic or OE-PGAP1 + miR-374b-5p mimic was measured by CCK-8 assay. Cell frequency (**C**) and apoptosis rate (**D**) of LPS-induced chondrocytes transfected with OE-NC, OE-PGAP1, OE-NC + NC mimic or OE-PGAP1 + miR-374b-5p mimic were calculated via flow cytometry analysis. **E** The protein levels of Caspase3, Caspase9, Cyclin A1, Cyclin B1 and Cyclin D2 in LPS-induced chondrocytes transfected with OE-NC, OE-PGAP1, OE-NC + NC mimic or OE-PGAP1 + miR-374b-5p mimic were determined via western blotting. **F** The levels of TNF-α, IL-1β and IL-6 in LPS-induced chondrocytes transfected with OE-NC, OE-PGAP1, OE-NC + NC mimic or OE-PGAP1 + miR-374b-5p mimic were determined via ELISA. *P < 0.05, **P < 0.01, ***P < 0.001 vs. LPS + OE-NC. #*P* < 0.05, ##*P* < 0.01, ###*P* < 0.001 vs. LPS + OE-PGAP1. Error bars represented as the means ± standard deviation (SD). Data were all analyzed by One-way ANOVA followed by Tukey's multiple comparisons test

Subsequently, the mRNA expression of PGAP1 in LPS-induced chondrocytes after transfection of OE-NC, OE-PGAP1, OE-NC + NC mimic or OE-PGAP1 + miR-374b-5p mimic was determined. As presented in Fig. 6A, PGAP1 expression was increased by OE-PGAP1 transfection but partially decreased when further transfected with miR-374b-5p, suggesting the successful transfection experiments. The mechanism between miR-374b-5p and PGAP1 in the progression of OA in vitro was then scrutinized. In the cellular level, ectopic expression of PGAP1 could enhance LPS-induced chondrocytes’ viability (Fig. 6B, *P * < 0.05), increase cell frequency in S phase (Fig. 6C, *P * < 0.001) and limit the apoptosis rate (Fig. 6D, *P * < 0.001). Apart from that, we further disclosed that PGAP1 overexpression repressed the protein levels of Caspase3, Caspase9, Cyclin A1, Cyclin B1 and Cyclin D2 (Fig. 6E, *P * < 0.001), and inhibited the levels of TNF-α, IL-1β and IL-6 (Fig. 6F, *P * < 0.001). However, upregulation of miR-374b-5p to some extent attenuated the promoting effect of PGAP1 overexpression on cell proliferation, and the repressive impacts on apoptosis and inflammation (Fig. 6B-F, *P * < 0.05).

### Indicators in an OA rat model

**Fig. 7:**
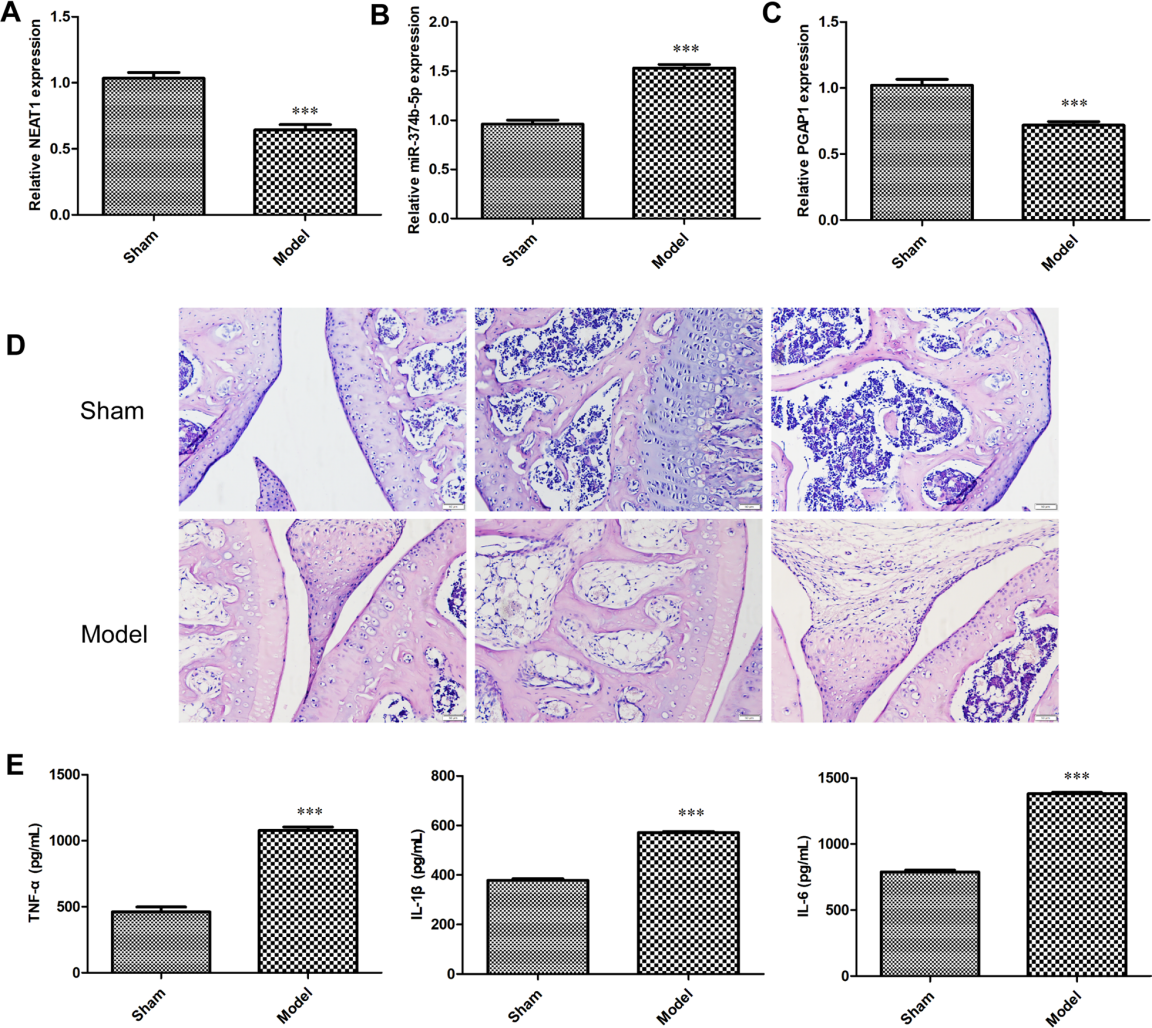
Indicators in an OA rat model. **A**–**C** The expression of NEAT1, miR-374b-5p and PGAP1 in the cartilage tissues of OA rats was detected via qRT-PCR. ***P < 0.001 vs. sham. **D** The pathological changes between the rats in the sham and model groups were observed via HE staining. **E** The levels of TNF-α, IL-1β and IL-6 in the serum of OA rats were determined via ELISA. ***P < 0.001 vs. sham. The animal experiments were performed in 5 rats in each group. Error bars represented as the means ± standard deviation (SD). Data were all analyzed by Student’s *t*-test

An OA rat model was established in this report. As illustrated in Fig. 7A–C, we found that, the expression of NEAT1 and PGAP1 was reduced, while miR-374b-5p expression was increased in OA cartilage tissues of rats (*P* < 0.001). Moreover, it was shown that compared with the sham group, the joint space in the model group was narrowed and hyaline cartilage was decreased. Meanwhile, calcified cartilage was increased and macrophage hyperplasia was observed (Fig. 7D). Additionally, the serum levels of TNF-α, IL-1β and IL-6 in the model group were overtly increased compared to those of sham group (Fig. 7E, *P * < 0.001).

## Discussion

OA is a common chronic joint disease that develops due to aging, and as life expectancy of the global population rises, the number of OA patients will increase sharply [40]. Globally, approximately 50% of geriatric population (over 60 years old) suffers from OA [41]. It has been estimated that there are over 43 million OA patients worldwide in 2002 and this number is expected to keep increasing in the next several decades [42]. Therefore, exploring effective therapies for OA is important for reduce this heavy burden on public health. In the current work, we have indicated a novel mechanism to regulate chondrocyte apoptosis and inflammation in OA progression, suggesting that pronounced downregulation of lncRNA NEAT1 can interact with miR-374b-5p/PGAP1 axis to accelerate the development of OA via promoting chondrocyte apoptosis and inflammation.

Accumulating investigations have manifested that some downregulated lncRNAs such as CAIF [43], DILC [44], and MIR4435-2HG [45] can distinguish OA patients from the healthy individuals and exert substantially stimulating impacts on the development of OA. Herein, pronounced downregulation of NEAT1 was also observed in OA patients. Additionally, OA cell model and rat model were established in this study and we demonstrated that NEAT1 was also lowly expressed in LPS-induced chondrocytes and OA rats. These results evidenced that the reduced expression of NEAT1 may serve as a pathogenic factor in the progression of OA. Therefore, we speculated that NEAT1 may be a therapeutic target for OA in clinic. Additionally, the aberrant expression levels of lncRNAs have been ascertained to be referred to the biological processes of OA chondrocytes including proliferation, cell cycle and apoptosis. For instance, loss of MIR4435-2HG has restrictive influences on the proliferative capacities of chondrocytes [45]. HOTAIRM1-1 silencing dramatically limits the viability of chondrocytes and induces apoptosis overtly [16]. Deficiency of CAIF not only distinctly accelerates the apoptosis of LPS-induced chondrocytes, but also promotes the secretion of IL-6 [43]. It is acknowledged that siRNAs selectively target a gene and silence it, inhibiting the expression of the gene that determines the pathology under study. Numerous siRNAs have been widely used to regulate the physiopathology of musculoskeletal diseases including tendon injuries and rheumatoid arthritis [46, 47]. On the one hand, they can be used to accurately determine the genes and molecules involved in musculoskeletal diseases; on the other, they can be used as a target therapy [47]. In the current work, we designed a siRNA to specifically suppress expression of NEAT1 and found that the silenced NEAT1 suppressed chondrocytes viability, led to the G1/S arrest and induced apoptosis. Not only that, it also elevated the levels of Caspase3, Caspase9, Cyclin A1, Cyclin B1 and Cyclin D2, and facilitated the secretion of TNF-α, IL-1β and IL-6. Our results lend credence to the previous research conducted by Wang et al. indicating that NEAT1 is lowly expressed in OA cartilage, while its knocking-down remarkably represses chondrocyte growth, elevates the apoptosis rate and increase the abundance of inflammatory cytokines [48]. We therefore believed that NEAT1 is a crucial lncRNA in the onset and development of OA, while knockdown of NEAT1 has distinctly promoting impacts on OA progression via inhibiting cell growth, promoting apoptosis and inducing the secretion of inflammatory cytokines. In clinical application, we could determine whether NEAT1 is involved in OA progression by measuring chondrocytes loss and inflammatory cytokine levels in OA, thereby treating OA in a targeted manner.

It is well-known that miRNAs, as a type of ncRNA molecules with 21–25-nt in length, possess transcriptional repression functions through target the corresponding mRNAs [49, 50]. The therapeutic potential of miRNA in musculoskeletal diseases such as tendon injuries and rheumatoid arthritis have been widely reported [51, 52]. Some have been validated in clinically relevant animal models, and others are in the preclinical development phase. Additionally, multiple miRNAs are also found to take prominent roles in the physiopathology, diagnosis and therapeutic potential of OA, as the biotechnology developed [53]. High expression levels of miR-10a-5p are closely associated with then enhanced apoptosis rate and worse cartilage surfaces [54]. The cell viability of IL-1β-induced chondrocytes with upregulated miR-216b is relatively lower [55]. It has been indicated the pronounced increased miR-590-5p in IL-1β-induced chondrocytes, and its overexpression is confirmed to suppress cell viability and promote inflammatory reactions [56]. For miR-374b-5p, there are also various reports implying its pro-apoptotic role in cancers, such as in non-small cell lung [57], pancreatic [58] and bladder [59] cancers. Meanwhile, miR-374b-5p has been confirmed to play a pro-inflammatory role in some systemic autoinflammatory diseases [60]. In the present investigation, we found high expression level of miR-374b-5p in OA patients, OA rats and LPS-induced chondrocytes. Moreover, in this study, we also showed introduction of miR-374b-5p mimic further potentiated the apoptosis rate and inflammatory responses in LPS-induced chondrocytes. These results implied that miR-374b-5p may be a feasible diagnostic biomarker for patients with OA. Some previous studies have displayed that lncRNA-mediated ceRNA network is deeply taken part in the onset and development of OA [61, 62]. Combined the biological functions of NEAT1 and miR-374b-5p in OA progression, we therefore speculated that NEAT1 may serve as the ceRNA of miR-374b-5p in the development of OA. As expected, NEAT1 could directly bind to miR-374b-5p in this investigation. Hence, we believed that NEAT1-mediated miR-374b-5p network synergistically aggravates the progression of OA.

PGAP1 is involved in regulating OA and is lowly expressed in LPS-treated chondrocytes and OA tissues [36]. Herein, we demonstrated a reduction in PGAP1 in OA patients, OA rats and LPS-induced chondrocytes. These data to some extent suggested that PGAP1 may act as an anti-osteoarthritis role in the development of OA. The results that PGAP1 overexpression noticeably inhibited apoptosis and inflammation in LPS-induced chondrocytes have validated this assumption. There is a recent study indicating that a lncRNA/miRNA/mRNA network may mediate the progression of OA [61]. We speculated that there may be a direct target gene modulated by NEAT1-miR-374b-5p in this investigation and this target gene was PGAP1. As expected, we evidenced that PGAP1 was a target gene of miR-374b-5p and it could be also positively modulated by NEAT1. Additionally, we further demonstrated that miR-374b-5p upregulation partially attenuated the repressive effects of PGAP1 overexpression on apoptosis and inflammation in LPS-induced chondrocytes. Collectively, we believed that downregulation of NEAT1 can induce the development of OA through promoting chondrocyte apoptosis and inflammation via miR-374b-5p/PGAP1 axis.

## Conclusion

To be concluded, the current work to some extent highlights a novel mechanism hidden in OA pathogenesis, indicating that downregulation of NEAT1 can interact miR-374b-5p/PGAP1 axis to promote chondrocyte apoptosis and inflammation, thereby accelerating the development of OA. The present study demonstrates that the NEAT1/miR-374b-5p/PGAP1 axis is essential in OA progression, pointing to act as a potential therapeutic target for OA in clinic. However, this study did not confirm the detailed mechanism among NEAT1, miR-374b-5p and PGAP1 in animal models, which may be a limitation of the present study. Further experiments will be performed to elucidate these issues in future.

## Data Availability

The datasets analyzed during the current study are available from the corresponding author on reasonable request.

## Abbreviations

OA: Osteoarthritis
LncRNA: Long noncoding RNA
lincRNA: Long intergenic noncoding RNA
NEAT1: Nuclear paraspeckle assembly transcript 1
XHOTAIRM1-1: Small nucleolar RNA host gene 7
SNHG7: Small nucleolar RNA host gene 7
CAIF: Cardiac autophagy inhibitory factor
DILC: Downregulated in liver cancer stem cells
MIR4435-2HG: MicroRNA 4435–2 host gene
miRNA: MicroRNA
ceRNA: Competing endogenous RNA
PGAP1: Post-GPI attachment to protein
LPS: Lipopolysaccharide
IL-6: Interleukin-6
TNF-α: Tumor necrosis factor-alpha
ECL: Enhanced chemiluminescence
HE: Hematoxylin and eosin
MUT: Mutant type
WT: Wild type
NC: Negative control
